# Age-dependent determinants of infectious complications profile in children and adults after hematopoietic cell transplantation: lesson from the nationwide study

**DOI:** 10.1007/s00277-019-03755-2

**Published:** 2019-07-18

**Authors:** Krzysztof Czyżewski, Jan Styczyński, Sebastian Giebel, Jowita Frączkiewicz, Małgorzata Salamonowicz, Olga Zając-Spychala, Agnieszka Zaucha-Prażmo, Joanna Drozd-Sokołowska, Anna Waszczuk-Gajda, Jarosław Dybko, Joanna Mańko, Patrycja Zalas-Więcek, Przemysław Gałązka, Mariusz Wysocki, Jerzy Kowalczyk, Jacek Wachowiak, Jolanta Goździk, Grzegorz W Basak, Krzysztof Kałwak, Monika Adamska, Marek Hus, Agnieszka Piekarska, Alicja Sadowska-Klasa, Patrycja Mensah-Glanowska, Sławomira Kyrcz-Krzemień, Monika Biernat, Agnieszka Wierzbowska, Piotr Rzepecki, Agnieszka Tomaszewska, Kazimierz Hałaburda, Lidia Gil, for Polish Society of Pediatric Oncology and Hematology and Polish Society of Hematology and Blood Transfusion

**Affiliations:** 1 Department of Pediatric Hematology and Oncology, Collegium Medicum, Nicolaus Copernicus University Torun, ul. Sklodowskiej-Curie 9, 85-094 Bydgoszcz, Poland; 2 Department of Hematology, Cancer Center and Institute of Oncology, Gliwice, Poland; 3 Department of Pediatric Transplantology, Hematology and Oncology, Medical University, Wroclaw, Poland; 4 Department of Pediatric Hematology, Oncology and Transplantology, Medical University, Poznan, Poland; 5 Department of Pediatric Hematology, Oncology and Transplantology, Medical University, Lublin, Poland; 6 Department of Hematology, Medical University, Warsaw, Poland; 7 Department of Hematology, Medical University, Wroclaw, Poland; 8 Present Address: Department and Clinic of Internal and Occupational Diseases, Hypertension and Clinical Oncology, Medical University, Wroclaw, Poland; 9 Department of Hematology, Medical University, Lublin, Poland; 10 Department of Microbiology, Collegium Medicum, Nicolaus Copernicus University Torun, Bydgoszcz, Poland; 11 Department of Pediatric Surgery, Collegium Medicum, Nicolaus Copernicus University Torun, Bydgoszcz, Poland; 12 Department of Pediatric Transplantology, Clinical Immunology and Transplantology, Collegium Medicum, Jagiellonian University, Krakow, Poland; 13 Department of Hematology, Poznan University of Medical Sciences, Poznan, Poland; 14 Department of Hematology, Medical University, Gdansk, Poland; 15 Department of Hematology, Collegium Medicum, Jagiellonian University, Krakow, Poland; 16 Department of Hematology, Medical University of Silesia, Katowice, Poland; 17 Department of Hematology, Medical University, Lodz, Poland; 18 Department of Hematology, Military Institute of Medicine, Warsaw, Poland; 19 Department of Hematology, Institute of Hematology and Transfusion Medicine, Warsaw, Poland

**Keywords:** Hematopoietic cell transplantation, Children, Adults, Incidence, Outcome, Bacterial infections, Viral infections, Invasive fungal disease

## Abstract

Incidence and outcome of microbiologically documented bacterial/viral infections and invasive fungal disease (IFD) in children and adults after hematopoietic cell transplantation (HCT) were compared in 650 children and 3200 adults in multicenter cross-sectional nationwide study. Infections were diagnosed in 60.8% children and 35.0% adults, including respectively 69.1% and 63.5% allo-HCT, and 33.1% and 20.8% auto-HCT patients. The incidence of bacterial infections was higher in children (36.0% vs 27.6%; *p* < 0.0001). Infections with Gram-negative bacteria were more frequent than Gram-positives in adults (64.6% vs 44.8%; *p* < 0.0001). Outcome of bacterial infections was better in children (95.5% vs 91.4%; *p* = 0.0011). The IFD incidence (25.3% vs 6.3%; *p* < 0.0001) and outcome (88.0% vs 74.9%; *p* < 0.0001) were higher in children. The incidence of viral infections was higher in children after allo-HCT (56.3% vs 29.3%; *p* < 0.0001), and auto-HCT (6.6% vs 0.8%; *p* < 0.0001). Outcome of viral infections was better in children (98.6% vs 92.3%; *p* = 0.0096). Infection-related mortality was 7.8% in children and 18.4% in adults (*p* < 0.0001). No child after auto-HCT died of infection. Adult age, mismatched transplants, acute leukemia, chronic GVHD, CMV reactivation, infection with Gram-negatives, and duration of infection > 21 days were risk factors for death from infection. In conclusion, pediatric patients have 2.9-fold higher incidence and 2.5-fold better outcome of infections than adults after HCT.

## Introduction

Infections are a significant cause of morbidity, mortality, and resource utilization after hematopoietic cell transplantation (HCT) in children and adults. Bacterial infections both after allo- and auto-HCT are known to be associated with high mortality and have become a public health problem of major concern worldwide due to antibiotic resistance. Invasive fungal disease (IFD) remains an important cause of morbidity and mortality after allo-HCT. The incidence of IFD has been reported at 9% after allo-HCT with mortality up to 50% of patients, especially after alternative donor transplantations [1–3]. Most studies have reported a high rate of viral infection after allo-HCT but not auto-HCT. High viral infection risk after allo-HCT is likely related to the delayed immune reconstitution after transplantation [4]. Recent EBMT (European Society for Blood and Marrow Transplantation) analysis showed that infections are responsible for 21.6% of deaths after allo-HCT and 11.0% after auto-HCT in all age groups together; however, the risk, types, and outcome of infections varied between age groups [5].

Infections occur in up to 82% of children [6–8] and adults [9–11] after HCT; however, large multicenter studies on incidence and outcome of bacterial, fungal, and viral infections are lacking. So far also no direct simultaneous comparison was made between children and adults.

In this study, we compared the incidence, type, and outcome of infections in pediatric and adult HCT centers in Poland in multicenter cross-sectional nationwide study. We analyzed also risk factors for the incidence and outcome of infections in 650 children and 3200 adults who received HCT.

## Patients and methods

### Design of the study

All consecutive patients transplanted between 1.01.2012 and 31.12.2015 in 5/5 pediatric, and in 11/13, adult HCT Polish centers were included in the retrospective study. Bacterial, fungal, and viral infections were reported biannually by each center and data were analyzed centrally.

### Bacterial infections

Among bacterial infections, only microbiologically documented (MDI) episodes were considered. Colonizations were not included into this analysis. MDI were diagnosed as bloodstream, gut, urinary tract, respiratory tract (broncho-alveolar lavage), and skin and soft tissue infections. Bacteria were analyzed with attention to resistance profile, such as ESBL (extended-spectrum β-lactamases: bacteria producing extended-spectrum β-lactamases), AmpC (AmpC β-lactamases: bacteria producing chromosomal cephalosporinase AmpC type), KPC (*Klebsiella pneumoniae* carbapenemase, *Enterobacteriaceae* producing carbapenemase KPC type) [12], MRSA/MRSE (methicillin-resistant *Staphylococcus aureus* or *epidermidis*), or VRE (vancomycin-resistant enterococci). Multidrug resistant (MDR) bacteria denote resistance to at least two antibiotics used in empiric therapy or resistance to at least three of antibiotic classes [13, 14].

### Fungal infections

The diagnosis of IFD was made according to EORTC/MSG criteria as proven, probable, or possible [15–17]. Patients were screened with galactomannan test mainly during neutropenia or on the basis of clinically driven indications. Diagnostics for *Pneumocystis jiroveci* pneumonia (PjP) was performed in case of clinical indications.

### Viral infections

Viral infections were classified as episodic (diagnosed on the basis of clinical picture, and supplemented with appropriate tests) or latent (diagnosed at molecular level). The following viruses were detected by PCR analysis: adenovirus (ADV), polyoma BKV, cytomegalovirus (CMV), Epstein-Barr virus (EBV), human herpesvirus 6 (HHV-6), and community-acquired respiratory viruses (CARV) including influenza.

### Supportive therapy

Uniform, standard anti-infective prophylaxis has been applied for patients undergoing HCT. Prophylactic, empirical, preemptive, or targeted anti-infectious therapy was performed with various antibacterial, antiviral, and antifungal agents according to commonly accepted strategies [13, 14, 18–21].

### Prophylaxis of infections

Environmental prophylaxis was applied in all centers according to commonly accepted policy [22]. In children, antibacterial prophylaxis consisted of oral penicillin or second-generation cephalosporin (from day − 10, until neutrophil count > 1 × 10^9^/L or end of immunosuppressive treatment) and oral gentamicin used from the beginning of conditioning until hematological recovery. Children received antifungal prophylaxis with fluconazole; from 2014, posaconazole was used in case of graft versus host disease (GVHD) or in secondary prophylaxis. In children under age of 12 years, the drug was used off-label [17] and administered according to body weight, as shown by Welzen et al [23]. In adults during neutropenia, fluoroquinolones were used for antibacterial prophylaxis and fluconazole in antifungal prophylaxis together with regular screening of serum galactomannan and computed tomography (HRCT/CT) in case of suspected IFD. Both in children and adults, acyclovir was used in prophylaxis of HSV/VZV infection until 1 year post-transplant. Weekly screening for DNA-emia and preemptive treatment were performed for CMV and EBV reactivation. Prevention of PjP included cotrimoxazole after hematopoietic recovery until the end of immunosuppressive treatment. Commercial immunoglobulin preparations were given in case of decreased immunoglobulin concentration during the first month and then monthly until B cell function recovery. Most of children receiving myeloablative conditioning (MAC) were commenced on gut rest from the first 5 days after HCT and received total parenteral nutrition (TPN) until hematopoietic recovery.

### Types of transplants

Transplants were divided as autologous and allogeneic from matched sibling donors (MSD) or unrelated donors: matched (MUD) or mismatched (MMUD). Most patients who underwent MUD/MMUD-HCT received anti-thymocyte globulin (ATG) [24].

### Statistical analysis

For analysis of incidence, infectious event was defined as the diagnosis of a first specific infectious disorder. Categorical variables were compared with the chi-square test, non-categorical variables were compared with the Mann-Whitney *U* test. Odds ratio (OR) and confidence intervals (95%CI) were calculated for the difference in occurrence of infections in patients. Cumulative 2-year incidences of bacterial, fungal, or viral infections were calculated using competing risk analysis [25], starting from the day of transplant to the day of the first infection. Death was considered as the competing event. Outcome of infection was regarded as positive in case of survival from infection or negative in case of death from infection. Infection-related mortality (IRM) was defined as any death that occurred in the presence of infection, starting from the day of diagnosis of infection. Death from infection was diagnosed as of bacterial, fungal, or viral cause; however, in many cases of IRM, patients suffered from multiple infections, and clinically the most symptomatic infection was regarded as the primary cause of death. In case of relapse and progression of malignancy, this was regarded as the primary cause of death, regardless of concomitant infection. The Kaplan-Meier method was used to determine IRM, counting from the day of diagnosis of infection. The relationship between the binary outcome, infection incidence, or death from infection, and other variables, regarded as risk factors, were analyzed using multivariate logistic regression: hazard risk (HR) and 95%CI were calculated for each factor. All reported *p* values are two-sided; *p* < 0.05 was considered as statistically significant.

## Results

### Overall characteristics of infections

A total number of 395/650 (60.8%) children and 1120/3200 (35.0%) adults (OR = 2.9, 95%CI = 2.0–3.6; *p* < 0.0001) were diagnosed for bacterial/viral MDI or IFD, including 345/499 (69.1%) and 676/1070 (63.5%) patients, respectively, after allo-HCT, while 50/151 (33.1%) and 444/2130 (20.8%) respectively, patients after auto-HCT. Patient characteristics and number of infections are shown in Table 1.

**Table 1:** Characteristics of patients

Characteristics	Children	Adults	*p*
All patients			
Total	650	3200	
Allo-HCT	499 (76.8%)	1070 (33.4%)	< 0.0001
Auto-HCT	151 (23.2%)	2130 (66.6%)	< 0.0001
Acute lymphoblastic leukemia (ALL)	153 (23.2%)	211 (6.6%)	< 0.0001
Acute myeloid leukemia (AML)	98 (15.1%)	472 (14.8%)	0.8306
Myelodysplastic syndromes (MDS)	32 (4.9%)	79 (2.5%)	0.0006
Non-Hodgkin lymphoma (NHL)/Hodgkin lymphoma (HD)	50 (7.7%)	803 (25.1%)	< 0.0001
Severe aplastic anemia (SAA)/bone marrow failure (BMF)	75 (11.5%)	58 (1.8%)	< 0.0001
Other			
Primary immunodeficiencies (PID)	75 (11.5%)		
Neuroblastoma (NBL)	49 (7.5%)		
Ewing sarcoma (ES)	13 (2.0%)		
Multiple myeloma (MM)		1308 (40.9%)	
Chronic myeloid leukemia (CML)/myeloproliferative neoplasms (MPN)		119 (3.8%)	
Other	43 (6.6%)	122 (3.8%)	
Patients with infections			
Total number of patients with infections	Children (*n* = 395)	Adults (*n* = 1120)	
Sex: male/female	230 (58.2%)/165 (41.8%)	634 (56.6%)/486 (43.4%)	0.5758
Age at HCT: median (range) [years]	7.2 (0.1–18.0)	50.1 (18.0–77.8)	< 0.0001
Donor type			< 0.0001
Sibling	97 (24.6)	224 (20.0)	
Mismatched related	4 (1.0)	2 (0.2)	
Unrelated	244 (61.7)	450 (40.2)	
Autologous	50 (12.7)	444 (39.6)	
Stem cell source			< 0.0001
Peripheral blood (PB)	243 (61.5)	1058 (94.5)	
Bone marrow (BM)	149 (37.7)	62 (5.5)	
Cord blood (CB)	3 (0.8)	0 (0.0)	
TBI given: yes/no	64 (16.2%)/331 (83.8%)	170 (15.2%)/950 (84.8%)	0.6282
Reduced intensity/standard conditioning	71 (18.0%)/324 (82.0%)	157 (14.0%)/963 (86.0%)	0.0585
Acute GVHD: yes/no (including auto-HCT)	92 (23.3%)/303 (76.7%)	166 (14.8%)/954 (85.2%)	0.0001
Neutrophil engraftment by day + 100: yes/no	378 (95.7%)/17 (4.3%)	1067 (95.3%)/53 (4.7%)	0.7273
Time to neutrophil engraftment: median (range) [days]	14.0 (8–51)	16.0 (6–60)	< 0.0001
Follow-up after HCT: median (range) [months]	21 (0–48)	19 (0–41)	0.6924

Legend: *HCT*, hematopoietic stem cell transplantation; *TBI*, total body irradiation; *GVHD*, graft versus host disease

Total number of infectious episodes was 3180, including 1399 in children (2.15 per patient) and 1781 in adults (0.56 per patient) (*p* < 0.0001). Also respective numbers of infections per patient were higher in children for bacterial (0.88 vs 0.37; *p* < 0.0001), fungal (0.38 vs 0.06; *p* < 0.0001), and viral (0.89 vs 0.13; *p* < 0.0001) episodes.

### Bacterial infections

#### Incidence

**Fig. 1:**
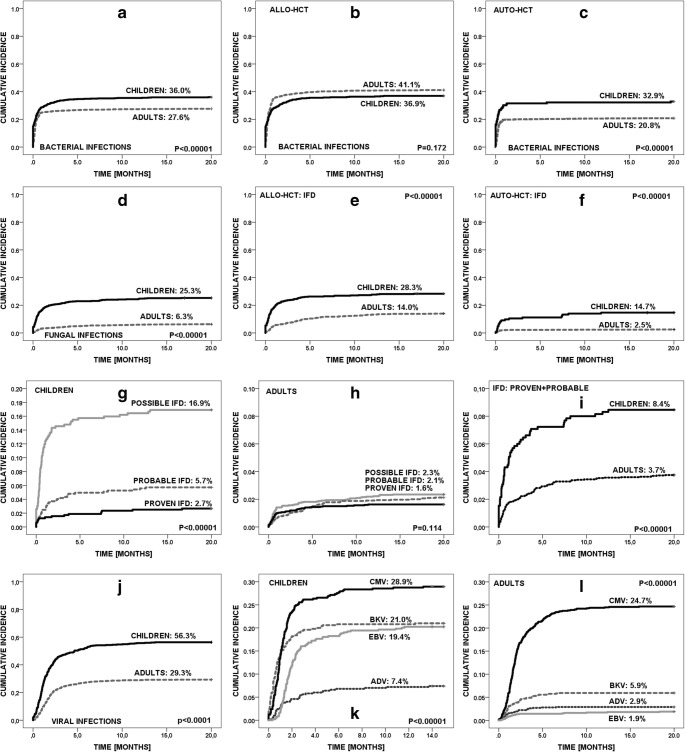
Incidence of infections. **a** Total, **b** allo-, and **c** auto-HCT bacterial infections. **d** Total, **e** allo-, and **f** auto-HCT fungal infections. **g** Proven, **h** probable, and **i** possible IFD. **j** Total viral infections. **k**, **l** CMV, BKV, EBV, and ADV infections in children and adult

The 2-year incidence of bacterial infections was 36.0% for children and 27.6% for adult patients (*p* < 0.0001), including allo-HCTs (36.9% vs 41.1%, ns), and auto-HCTs (32.9% vs 20.8%; *p* < 0.0001) (Fig. 1a–c). These numbers were however comparable for specific primary diseases including acute lymphoblastic leukemia (ALL), acute myeloblastic leukemia/myelodysplastic syndrome (AML/MDS), non-Hodgkin lymphoma/Hodgkin lymphoma (NHL/HD), and severe aplastic anemia (SAA). Only 12.9% adults with multiple myeloma (MM) after auto-HCT had bacterial infections (Table 2).

**Table 2:** Frequency of infections

Parameter	Children	Adults	OR	*p*
Bacterial infections				
Total	36.0% (234/650)	27.6% (882/3200)	1.5 (1.2–1.8)	< 0.0001
Allo-HCT	36.9% (184/499)	41.1% (440/1070)	0.8 (0.7–1.04)	0.1093
Auto-HCT	32.9% (50/151)	20.8% (444/2135)	1.9 (1.3–3.7)	0.0004
ALL	45.1% (69/153)	42.6% (90/211)	1.1 (0.7–1.6)	0.6426
AML	38.8% (38/98)	38.8% (183/472)	1.0 (0.6–1.5)	0.9752
MDS	34.8% (11/32)	38.0% (30/79)	0.9 (0.4–2.0)	0.7218
NHL/HD	28.0% (14/50)	19.7% (158/803)	1.6 (0.8–3.0)	0.1546
SAA/BMF	25.3% (19/75)	19.0% (11/58)	1.4 (0.6–3.3)	0.3835
Other				
PID	37.3% (28/75)	–	ND	ND
NBL	44.9% (44/98)	–	ND	ND
ES	30.8% (8/26)	–	ND	ND
MM	–	12.9% (169/1308)	ND	ND
Fungal infections				
Total	25.3% (163/650)	6.3% (183/3200)	5.5 (4.3–6.9)	< 0.0001
Possible	16.9% (98/650)	2.3% (91/3200)	6.1 (4.5–8.1)	< 0.0001
Probable	5.7% (42/650)	2.1% (49/3200)	4.4 (2.9–6.8)	< 0.0001
Proven	2.7% (31/650)	1.6% (43/3200)	3.7 (2.3–5.9)	< 0.0001
Allo-HCT	28.3% (142/499)	14.0% (132/1070)	2.8 (2.1–3.7)	< 0.0001
Auto-HCT	14.7% (21/151)	2.5% (51/2135)	6.6 (3.8–11.3)	< 0.0001
ALL	29.4% (45/153)	11.3% (24/211)	3.2 (1.8–5.6)	< 0.0001
AML/MDS	41.2% (47/114)	13.2% (73/551)	4.6 (2.9–7.2)	< 0.0001
Viral infections				
Auto-HCT	6.6% (10/151)	0.8% (17/2135)	8.8 (4.0–19.6)	< 0.0001
Allo-HCT	56.3% (281/499)	29.3% (314/1070)	3.1 (2.5–3.8)	< 0.0001
Multiple viral infections	34.5% (97/281)	27.1% (85/314)	1.4 (1.0–2.0)	0.0498
Adenovirus (ADV)	7.4% (37/499)	2.9% (31/1070)	3.5 (2.0–6.0)	< 0.0001
Polyoma BKV (BKV)	21.0% (104/499)	5.2% (63/1070)	5.2 (3.7–7.5)	< 0.0001
Cytomegalovirus (CMV)	28.9% (144/499)	24.7% (264/1070)	1.7 (1.3–2.1)	< 0.0001
Epstein-Barr virus (EBV)	19.4% (97/499)	1.9% (20/1070)	15.7 (9.2–26.1)	< 0.0001
Influenza virus (INFL)	2.2% (11/499)	0.5% (5/1070)	4.8 (1.6–13.9)	0.0014
Other				
Varicella-zoster virus (VZV)	1.8% (9)	0.3% (3)	ND	ND
HHV6	1.2% (6)	0.4% (4)	ND	ND
Respiratory syncytial virus (RSV)	0.8% (4)	0.2% (2)	ND	ND
Parainfluenza virus (PIF)	0.4% (2)	0	ND	ND
Metapneumovirus (MPV)	0.2% (1)	0	ND	ND
Rotavirus/norovirus	8.4% (42)	0.3% (3)	ND	ND

Legend: *HCT*, hematopoietic stem cell transplantation; *AML*, acute myeloid leukemia; *ALL*, acute lymphoblastic leukemia; *MDS*, myelodysplastic syndromes; *NHL*, non-Hodgkin lymphoma (NHL); *HD*, Hodgkin lymphoma; *SAA*, severe aplastic anemia (SAA); *BMF*, bone marrow failure; *PID*, primary immunodeficiencies; *NBL*, neuroblastoma; *ES*, Ewing sarcoma; *MM*, multiple myeloma; *ND*, not done

Infections with Gram-negative bacteria were more frequent than Gram-positive in adults (64.6%), but not in children (44.8%). The difference was highly significant (*p* < 0.0001; OR = 2.3, 95%CI = 1.8–2.7). The frequency of G-negative bacteria with MDR phenotype was 67.8% (158/233) in children and 47.9% (404/843) in adults (OR = 2.3; 95%CI = 1.7–3.1, *p* < 0.0001). The frequency of G-positive bacteria with MDR phenotype was 43.6% (125/287) in children and 40.1% (185/461) in adults (OR = 1.1; ns). Detailed etiology of G-negative and G-positive infections is presented in Tables 3 and 4.

**Table 3:** Distribution and multidrug resistance (MDR) phenotypes of isolated Gram-negative species. The total number of MDR strains contains all types of resistance, including coexistence of mechanisms

Pathogen	Children					Adults				
	Total	MDR	ESBL	AmpC	Other	Total	MDR	ESBL	AmpC	Other
Total	233	158 (67.8%)	153 (65.7%)	5 (2.1%)	4	843	404 (47.9%)	378 (44.8%)	70 (8.3%)	41 (4.8%)
*Escherichia coli*	58	44 (75.9%)	44 (75.9%)	–	–	242	75 (31.0%)	75 (31.0%)	7 (2.9%)	–
*Klebsiella pneumoniae*	38	32 (84.2%)	29 (76.3%)	–	–	274	181 (66.1%)	181 (66.1%)	40 (14.6%)	14 (5.1%)
*Enterobacter cloacae*	56	52 (92.9%)	52 (92.9%)	3 (5.4%)	–	115	79 (68.7%)	70 (60.9%)	21 (18.3%)	10 (8.7%)
*Pseudomonas aeruginosa*	22	1 (4.5%)	–	–	–	74	36 (48.6%)	22 (29.7%)	2 (2.7%)	14 (18.9%)
*Klebsiella oxytoca*	13	12 (92.3%)	12 (92.3%)	1 (7.7%)	–	17	7 (41.2%)	7 (41.2%)	–	–
*Citrobacter freundii*	8	4 (50.0%)	3 (37.5%)	1 (12.5%)	–	7	5 (71.4%)	5 (71.4%)	–	–
*Acinetobacter junii*	1	–	–	–	–	19	4 (21.1%)	4 (21.1%)	–	–
*Proteus mirabilis*	2	2 (100%)	2 (100%)	–	–	19	4 (21.1%)	4 (21.1%)	–	–
Stenotrophomonas maltophilia*	11	–	–	–	–	29	–	–	–	–
*Salmonella enteritidis*	–	–	–	–	–	6	–	–	–	–
*Enterobacter faecium*	2	–	–	–	–	13	–	–	–	–
*Enterobacter aerogenes*	8	8 (100%)	8 (100%0	–	–	3	2 (66.7%)	2 (66.7%)	–	–
*Klebsiella* spp.	3	1 (33.3%)	1 (33.3%)	–	–	–	–	–	–	–
*Morganella morganii*	1	1 (100%)	1 (100%)	–	–	7	3 (42.9%)	2 (28.6%)	–	1 (14.3%)
*Enterobacter* spp.	2	–	–	–	–	5	1 (20.0%)	1 (20.0%)	–	–
*Serratia* spp.	–	–	–	–	–	10	7 (70.0%)	5 (50.0%)	–	2 (20.0%)
Other species	6	–	–	–	–	–	–	–	–	–

* *Stenotrophomonas maltophilia* is inherently resistant to most antibiotics except for cotrimoxazole (trimethoprim-sulfamethoxazole) and ticarcillin-clavulanate

**Table 4:** Distribution and multidrug resistance (MDR) phenotypes of isolated Gram-positive species. The total number of MDR strains contains all types of resistance, including coexistence of mechanisms

Pathogen	Children					Adults				
	Total	MDR	MRS	VRE	Other	Total	MDR	MRS	VRE	Other
Total	287	125 (43.6%)	56 (19.5%)	49 (17.1%)	34 (11.8%)	461	185 (40.1%)	111 (24.1%)	59 (12.8%)	15 (3.3%)
*Clostridium difficile*	81	–	–	–	–	92	–	–	–	–
*Staphylococcus epidermidis*	51	36 (70.6%)	36 (70.6%)	–	–	129	74 (57.4%)	74 (57.4%)	–	–
*Staphylococcus aureus*	16	5	5	–	–	20	15 (75.0%)	15 (75.0%)	–	–
*Staphylococcus hominis*	9	–	–	–	–	25	4 (16.0%)	4 (16.0%)	–	–
*Enterococcus faecium*	86	69 (80.2%)	–	49 (57.0%)	34 (39.5%)	53	41 (77.4%)	–	26 (47.2%)	15 (28.3%)
*Staphylococcus haemolyticus*	18	5 (27.8%)	5 (27.8%)	–	–	33	10 (30.3%)	10 (30.3%)	–	–
*Enterococcus faecalis*	4	–	–	–	–	67	33 (49.3%)	–	33 (49.3%)	–
*Staphylococcus* spp.	2	–	–	–	–	19	8 (42.1%)	8 (42.1%)	–	–
*Streptococcus mitis*	1	–	–	–	–	6	–	–	–	–
*Streptococcus* spp.	3	–	–	–	–	–	–	–	–	–
*Staphylococcus warneri*	2	–	–	–	–	–	–	–	–	–
*Corynebacterium* spp.	1	–	–	–	–	7	–	–	–	–
*Streptococcus pyogenes*	2	–	–	–	–	–	–	–	–	–
*Streptococcus pneumoniae*	3	–	–	–	–	2	–	–	–	–
*Streptococcus oralis*	2	–	–	–	–	3	–	–	–	–
*Micrococcus* spp.	–	–	–	–	–	5	–	–	–	–
Other species	6	–	–	–	–	–	–	–	–	–

#### Timing

Median time from the day of HCT to bacterial infection was 0.20 months (range − 0.2–20.6) in children and 0.23 months (range − 0.3–36.9) in adults. Median time of therapy of bacterial infection was 14 days (range 1–196; quartiles 10–21) in children and 9 days (range 1–36; quartiles 8–14) in adults (*p* < 0.0001).

#### Risk factors

In multivariate logistic analysis (Table 5), the risk of infections was higher after allo-HCT than auto-HCT (HR = 1.8; *p* < 0.001). In allo-HCT patients, the risk was higher in children (HR = 2.1; *p* < 0.001), in acute leukemia (HR = 1.6; *p* < 0.001), MUD vs MSD-HCT (HR = 1.6; *p* < 0.001), MMUD vs MSD-HCT (HR = 2.0; *p* < 0.001), MAC (myeloablative conditioning) vs RIC (reduced-intensity conditioning) (HR = 1.3; *p* < 0.001), late (> 21 days) hematological recovery (HR = 3.3; *p* < 0.001), acute GVHD before infection (HR = 1.7; *p* < 0.001), and chronic GVHD before infection (HR = 1.4; *p* = 0.014). In auto-HCT patients, the risk was higher in children (HR = 1.7; *p* < 0.001), and in patients with late (> 21 days) hematological recovery (HR = 2.8; *p* < 0.001). In patients with multiple myeloma (MM), the risk was lower in comparison to all other patients (HR = 0.7; *p* = 0.005).

**Table 5:** Multivariate analysis of risk factors for infection

Risk factor	Bacterial infection		Fungal infection		Viral infection	
	HR (95%CI)	*p*	HR (95%CI)	*p*	HR (95%CI)	*p*
Allo vs auto	2.9 (2.3–3.6)	< 0.001	5.8 (4.6–6.9)	0.021	16 (12–20)	< 0.001
Allo-HCT						
Children vs adults	2.1 (1.8–2.4)	< 0.001	3.9 (3.3–4.5)	< 0.001	1.3 (1.05–1.5)	0.010
Sex male vs female	1.1 (0.9–1.3)	0.065	0.9 (0.8–1.1)	0.614	0.9 (0.8–1.1)	0.180
Acute leukemia vs other	1.6 (1.4–1.8)	< 0.001	3.8 (3.1–4.5)	< 0.001	1.7 (1.4–2.1)	< 0.001
NHL/HD vs other	1.2 (0.8–1.6)	0.521	1.3 (0.8–1.8)	0.358	1.4 (0.7–2.0)	0.642
Haplo vs other	1.4 (0.8–1.9)	0.162	1.3 (0.7–2.0)	0.315	0.8 (0.3–1.3)	0.520
MUD vs MSD	1.6 (1.2–2.1)	< 0.001	1.5 (1.1–1.8)	0.013	2.0 (1.3–2.7)	< 0.001
MMUD vs MSD	2.0 (1.4–2.6)	< 0.001	2.5 (2.1–2.9)	< 0.001	3.3 (2.7–3.9)	< 0.001
BM vs PB	1.4 (1.1–1.7)	0.007	1.2 (0.8–1.7)	0.382	0.8 (0.5–1.1)	0.165
MAC vs RIC	1.3 (1.0–1.6)	0.042	1.2 (0.9–1.6)	0.068	1.8 (1.0–2.5)	0.050
TBI vs chemotherapy	1.2 (1.0–1.4)	0.055	1.3 (0.8–1.7)	0.215	1.1 (0.7–1.5)	0.386
ANC recovery: >D21 vs ≤D21 days	3.3 (2.6–4.1)	< 0.001	1.2 (0.7–1.9)	0.729	0.8 (0.5–1.1)	0.065
aGVHD before infection: yes vs no	1.7 (1.4–2.1)	< 0.001	1.5 (1.1–2.0)	0.021	1.5 (1.1–2.0)	< 0.001
cGVHD before infection: yes vs no	1.4 (1.1–1.6)	0.014	2.2 (1.8–2.5)	< 0.001	2.7 (2.3–3.1)	< 0.001
Auto-HCT						
Children vs adults	1.7 (1.3–2.1)	< 0.001	1.8 (1.1–2.6)	0.025	1.1 (0.6–1.7)	0.872
Sex male vs female	1.2 (0.8–1.5)	0.475	0.8 (0.3–1.5)	0.235	0.8 (0.6–1.1)	0.294
Acute leukemia vs other	1.8 (1.4–2.2)	< 0.001	1.7 (1.2–2.1)	0.004	1.5 (1.1–1.2.0)	0.005
NHL/HD vs other	1.0 (0.80–1.2)	0.922	0.9 (0.7–1.2)	0.689	1.1 (0.7–1.4)	0.745
MM vs other	0.7 (0.4–0.9)	0.005	0.6 (0.3–0.8)	< 0.001	0.5 (0.2–0.8)	< 0.001
ANC recovery: >D21 vs ≤D21 days	2.8 (2.1–3.5)	< 0.001	1.0 (0.5–1.5)	0.936	0.9 (0.5–1.3)	0.825

Legend: *HCT*, hematopoietic stem cell transplantation; *AML*, acute myeloid leukemia; *ALL*, acute lymphoblastic leukemia; *BM*, bone marrow; *PB*, peripheral blood; *TBI*, total body irradiation; *a/cGVHD*, acute/chronic graft versus host disease; *MUD*, matched unrelated donor; *MSD*, matched sibling donor; *MMUD*, mismatched unrelated donor; *MAC*, myeloablative conditioning; *RIC*, reduced intensity of conditioning; *NHL*, non-Hodgkin lymphoma (NHL); *HD*, Hodgkin lymphoma; *MM*, multiple myeloma; *ANC*, absolute neutrophil count; *D*, days

#### Outcome

**Fig. 2:**
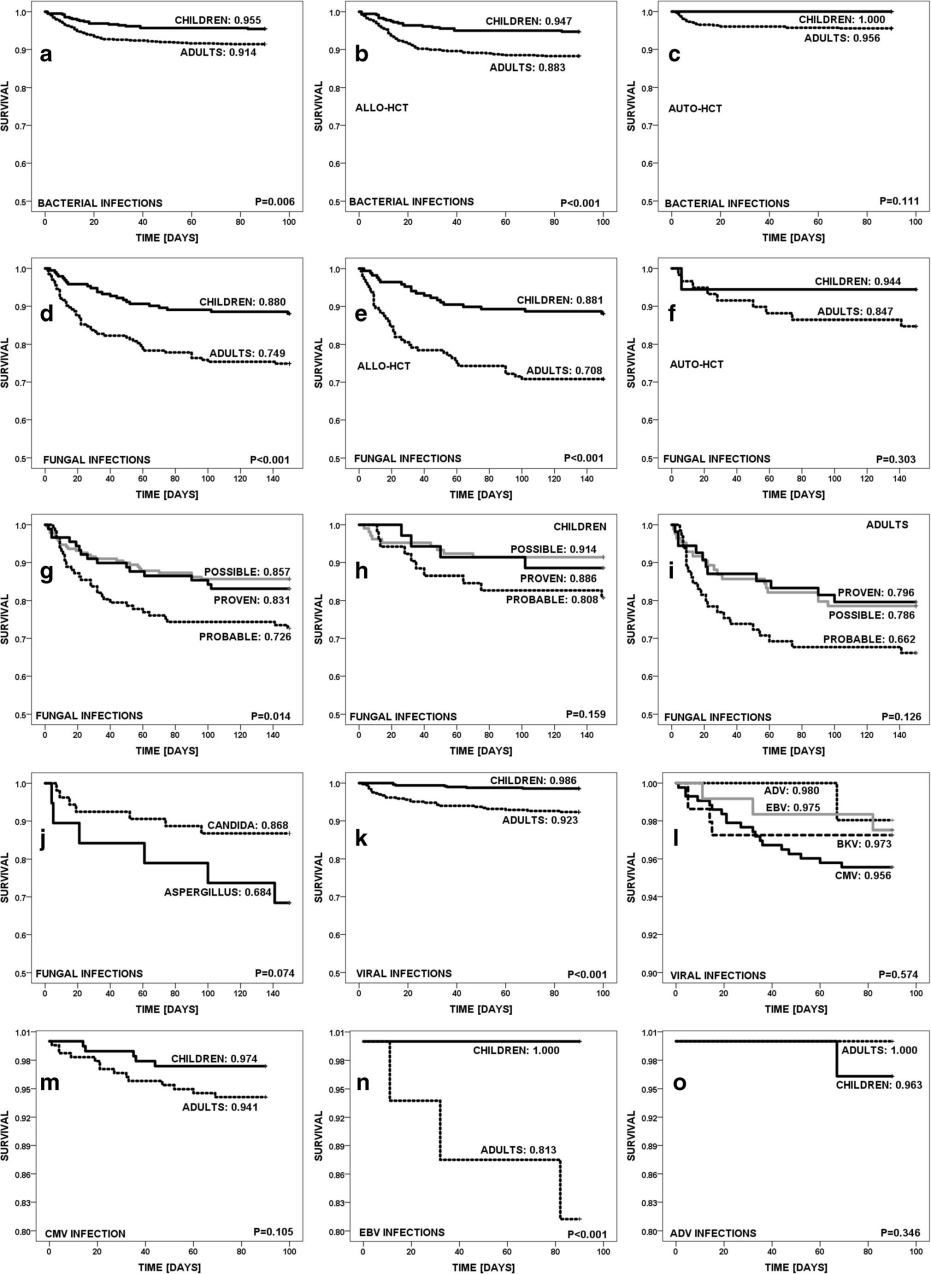
Outcome of infections. Bacterial infections **a** total, **b** allo-, and **c** auto-HCT. Fungal infections **d** total, **e** allo-, and **f** auto-HCT. With respect to level of IFD diagnosis **g** total, **h** children, and **i** adults. **j** Candida vs Aspergillus infections. Viral infections in **k** children vs adults. **l** CMV, BKV, EBV, and ADV infections. **m** CMV in children vs adults. **n** EBV in children vs adults. **o** ADV in children vs adults

Overall outcome of bacterial infections was positive in 95.5% of infections in children and in 91.4% in adults (OR = 3.2; 95%CI = 1.6–6.5; *p* = 0.0011). The outcome of infections was better in children both after allo- and auto-HCT (Fig. 2a–c).

### Fungal infections

#### Incidence

The 2-year incidence was 25.3% for children and 6.3% for adults (*p* < 0.001). It was higher both in children for allo-HCTs (28.3% vs 14.0%; *p* < 0.0001) and auto-HCTs (14.7% vs 2.5%; *p* < 0.0001) (Fig. 1d–f), regardless of the level of diagnosis: proven (2.7% vs 1.6%; *p* < 0.0001), probable (5.7% vs 2.1%; *p* < 0.0001), or possible IFD (16.9% vs 2.3%; *p* < 0.0001). At 2 years after HCT, incidences of proven/probable IFD were 8.4% and 3.7% (*p* < 0.0001) for children and adults, respectively (Fig. 1g–i). The frequency was higher both in pediatric patients with ALL (29.4% vs 11.3%; OR = 3.2, *p* < 0.0001) and AML/MDS (41.2% vs 13.2%; OR = 4.6, *p* < 0.0001), when compared to adults (Table 2). Additionally, two cases of PjP infections were diagnosed (one pediatric, one adult).

#### Identification of fungal species

Total number of identified proven fungal infections was 74 (31 in children and 43 in adults), including 31 (42%) aspergilloses (*A.* spp. 6 vs 14; *A. fumigatus* 5 vs 2; *A. flavus* 2 vs 2), 34 (46%) candidiases (*C. albicans* 9 vs 8; *C. glabrata* 3 vs 4; *C. krusei* 1 vs 1; *C. parapsilosis* 1 vs 0; *C. dublininsis* 0 vs 1; *C. guillerimondi* 0 vs 1; *C. kefyr* 0 vs 1), 5 (6.8%) mucormycoses (*Mucor* spp. 1 vs 3; *Rhizopus* spp. 1 vs 0), and 4 (5.2%) other species (*Fusarium* spp. 2 vs 1; *Cryptococcus* spp. 0 vs 1).

#### Timing

Median time from day of HCT to IFD was 0.9 months (range 0–19) in children and 0.7 months (range 0–20) in adults. Median time of therapy of IFD was 24 days (range 1–590; quartiles 12–47) in children and 10 days (range 1–406; quartiles 9–26) in adults (*p* < 0.0001).

#### Risk factors

In multivariate analysis, the risk of proven/probable IFD was higher after allo-HCT than auto-HCT (HR = 5.4; *p* < 0.001). In allo-HCT patients, the risk was higher in children than in adults (HR = 3.9; *p* < 0.001), in acute leukemia (HR = 3.8; *p* < 0.001), MUD vs MSD-HCT (HR = 1.5; *p* = 0.013), MMUD vs MSD-HCT (HR = 2.5; *p* < 0.001), late (> 21 days) hematological recovery (HR = 3.3; *p* < 0.001), acute GVHD before infection (HR = 1.5; *p* = 0.021), and chronic GVHD before infection (HR = 2.2; *p* < 0.001). In auto-HCT patients, the risk was higher in children than in adults (HR = 1.8; *p* = 0.025). Patients with MM were at lower risk of IFD in comparison to all other patients (HR = 0.6; *p* = 0.005) (Table 5).

#### Outcome

Overall outcome of IFD was positive in 88.0% of infections in children and in 74.9% in adults (OR = 2.1; 95%CI = 1.4–3.1; *p* < 0.0001). The outcome of IFD was better in children than adults both after allo- and auto-HCT, regardless of the level of IFD diagnosis: proven (88.6% vs 79.6%), probable (80.8% vs 66.2%), or possible (91.4% vs 78.6%) (Fig. 2d–j).

### Viral infections

#### Incidence

The 2-year incidence of viral infections was 56.3% for children and 29.3% for adults (*p* < 0.0001) after allo-HCT, and 6.6% vs 0.8% (*p* < 0.0001) after auto-HCT. The frequency was higher for CMV (28.9% vs 24.7%; OR = 1.7; *p* < 0.05), BKV (21.0% vs 5.9%; OR = 5.2; *p* < 0.0001), EBV (19.4% vs 1.9%; OR = 15.7; *p* < 0.0001), ADV (7.4% vs 2.9%; OR = 3.5; *p* < 0.0001), and influenza (2.2% vs 0.5%; OR = 4.8; *p* = 0.0038) (Table 2, Fig. 1j–l).

Viral infections after auto-HCT in children developed in 10 patients including 2 (1.3%) CMV, 1 (0.6%) influenza, 1 (0.6%) BKV, 1 (0.6%) ADV, and 5 (3.3%) rotavirus. Viral infections after auto-HCT in adults developed in 15 patients including 4 (0.18%) CMV, 5 (0.23%) influenza, 3 (0.14%) VZV, 2 (0.09%) HHV6, and 1 (0.04%) ADV.

#### Timing

Median time from day of HCT to viral infection was 1.4 months (range 0–19) in children and 1.6 months (range 0–21) in adults. Median time of therapy of viral infection was 13 days (range 0–168; quartiles 6–24) in children and 12 days (range 0–401; quartiles 1–28) in adults.

#### Risk factors

In multivariate analysis, the risk of infections was higher after allo-HCT (HR = 6.1; *p* < 0.001). In allo-HCT patients, the risk was higher in children (HR = 1.3; *p* = 0.010), in acute leukemia (HR = 1.7; *p* < 0.001), MUD vs MSD-HCT (HR = 2.0; *p* < 0.001), MMUD vs MSD-HCT (HR = 3.3; *p* < 0.001), MAC vs RIC (HR = 1.8; *p* = 0.050), acute GVHD before infection (HR = 1.5; *p* < 0.001), and chronic GVHD before infection (HR = 2.7; *p* = 0.014). Among auto-HCT patients, diagnosis of MM brought the lower risk of viral infections (HR = 0.5; *p* < 0.001) (Table 5).

#### Outcome

Overall outcome of viral infections was positive in 98.6% of infections in children and in 92.3% in adults (OR = 3.3; 95%CI = 1.2–8.7; *p* = 0.0096). The outcome of viral infections varied between children vs adults: CMV (97.4% vs 94.1%; *p* = 0.1), BKV (99.0% vs 93.9%; *p* = 0.075), EBV (100% vs 81.3%; *p* < 0.001), ADV (100% vs 96.3%; *p* = 0.3), and influenza (100% vs 70%; *p* = 0.5) (Fig. 1k–o).

### Deaths from infections

#### Frequencies

Overall, 237 patients died from infection, including 7.8% (31/395) children and 18.4% (206/1120) adults (OR = 0.4, 95%CI = 0.3–0.6; *p* < 0.0001). The distribution of deaths was different in children (35.5% bacterial, 48.4% fungal, 16.1% viral) than in adults (61.7% bacterial, 24.7% fungal, 13.6% viral).

#### Risk factors for death from infectious complications

In allo-HCT patients, in multivariate analysis, adults (HR = 3.3; *p* < 0.001), recipients of MMUD-HCT (HR = 3.8; *p* < 0.001), patients with acute leukemia (HR = 1.5; *p* = 0.023), chronic GVHD before infection (HR = 3.6; *p* = 0.014), CMV reactivation (HR = 1.4; *p* = 0.038), and in patients with duration of infection treatment > 21 days (HR = 1.4; *p* = 0.038) were associated with increased risk of IRM (Table 6). In separate analysis of patients with bacterial infections, the risk was higher in Gram-negative in comparison to Gram-positive infections (HR = 1.6; 95%CI = 1.1–2.1; *p* = 0.031).

**Table 6:** Multivariate analysis for risk factors for death from infection after HCT

Risk factor	HR (95%CI)	*p*
Allo vs auto-HCT	6.3 (5.2–7.6)	< 0.001
ALLO-HCT		
Adults vs children	3.3 (3.0–3.6)	< 0.001
Bacterial infection	1.1 (0.8–1.4)	0.527
Fungal (*p*/*p*) infection	1.6 (0.9–2.4)	0.082
Viral infection	1.5 (0.7–2.2)	0.392
CMV reactivation	1.4 (1.0–1.8)	0.038
Sex male vs female	1.1 (0.9–1.3)	0.962
Acute leukemia vs other	1.5 (1.1–1.9)	0.023
NHL/HD vs other	1.0 (0.8–1.2)	0.873
Haplo vs other	1.1 (0.8–1.3)	0.285
MUD/ vs MSD	1.3 (0.8–1.8)	0.259
MMFD vs MSD	3.8 (3.0–4.7)	< 0.001
BM vs PB	0.8 (0.3–1.4)	0.337
MAC vs RIC	1.1 (0.5–1.7)	0.872
TBI vs chemotherapy	1.1 (0.8–1.4)	0.498
aGVHD before infection: yes vs no	1.0 (0.5–1.6)	0.849
cGVHD before infection: yes vs no	3.6 (2.2–4.7)	< 0.001
ANC recovery: >D21 vs ≤D21 days	1.6 (0.8–2.5)	0.115
First infection: <D30 vs ≥D30 days	1.1 (0.4–1.6)	0.705
Treatment duration of infection: >D21 vs ≤D21 days	1.9 (1.1–2.7)	0.027
Auto-HCT		
Adults vs children	2.9 (1.5–4.8)	< 0.001
Bacterial infection	1.4 (0.7–2.2)	0.585
Fungal (*p*/*p*) infection	1.4 (0.7–2.3)	0.732
Viral infection	1.1 (0.2–1.9)	0.856
Sex male vs female	1.3 (0.8–1.9)	0.439
NHL/HD vs other	1.1 (0.3–1.9)	0.823
MM vs other	0.4 (0.1–0.8)	< 0.001
ANC recovery: >D21 vs ≤D21 days	1.3 (0.9–1.8)	0.092
First infection: <D30 vs ≥D30 days	1.1 (0.6–1.7)	0.774
Treatment duration of infection: >D21 vs ≤D21 days	1.7 (1.2–2.5)	< 0.001

Legend. *HR*, hazard ratio; *AML*, acute myeloid leukemia; *ALL*, acute lymphoblastic leukemia; *AL*, acute leukemia; *HCT*, hematopoietic cell transplantation; *BM*, bone marrow; *PB*, peripheral blood; *CMV*, cytomegalovirus; *TBI*, total body irradiation; *a/cGVHD*, acute/chronic graft versus host disease; *MUD*, matched unrelated donor; *MSD*, matched sibling donor; *MMUD*, mismatched unrelated donor; *MAC*, myeloablative conditioning; *RIC*, reduced intensity of conditioning; *NHL*, non-Hodgkin lymphoma (NHL); *HD*, Hodgkin lymphoma; *MM*, multiple myeloma; *ANC*, absolute neutrophil count; *D*, days

Among auto-HCT patients, no child died of infection. Among adults, the risk of death was higher if duration of treatment of infection was > 21 days (HR = 1.7; *p* < 0.001) (Table 6). In patients with MM, the risk was lower (HR = 0.4; *p* < 0.001). In separate analysis of patients with bacterial infections, there was a trend towards higher IRM in Gram-negative vs Gram-positive infections (HR = 1.8; 95%CI = 0.9–2.7; *p* = 0.086).

## Discussion

In this study for the first time ever, simultaneous analysis and comparison of epidemiology and outcome of bacterial, fungal, and viral infections in a large cohorts of children and adults after HCT in a multicenter cross-sectional nationwide study were performed. Both groups largely differed in terms of distribution of primary diseases and their treatment, types of preparative regimens, and types of transplantation. Although both pediatric and adult transplant centers used generally very similar strategy of anti-infective management [18], some differences between these settings existed, as pediatric centers used many off-label compounds. In this study, we analyzed patients over a period of 4 years, when anti-infective prophylaxis and treatment did not change substantially in both pediatric and adult centers.

Bacterial infections occurred mainly during neutropenic, pre-engraftment phase. In adults, Gram-negative bacteria were more often documented, while in children Gram-positive species. The rate of MDR Gram-negative strains was higher in pediatric than in adult centers, while the rate of Gram-positive MDR was comparable in these cohorts. Our results indicate the shift of prevalence from Gram-positive to Gram-negative bacteria in a population of adult hematology patients and increasing incidence of MDR bacteria, especially Gram-negative [26, 27]. It is debatable, if use of quinolones in adults or oral gentamycin in children have possible negative impact in the selection of resistant gut microbiome [27]. We confirmed that irrespectively to age, transplant performed from alternative donor and prolonged neutropenia were independent risk factors for the development of bacterial infection [28]. The differences in bacterial epidemiology between children and adults resulted in differences in outcome of bacterial infections in these two cohorts, with a higher risk for death related to Gram-negative bacteria. High rate in bacterial infections was found for typical pediatric primary diagnoses like primary immunodeficiencies, neuroblastoma, and Ewing sarcoma; and opposite, in adulthood disease multiple myeloma, the rate was much lower and reached 12.9%.

Fungal infections were much more frequently diagnosed in children, regardless of the level of diagnosis; however, it was predominant for possible IFD. It reflects “real-life” pediatric strategy of reducing invasive diagnostics in children. It seems that lower incidence of IFD in adults might result from general strategy of protective environment in transplant setting and the prophylactic use of posaconazole during intensive chemotherapy in AML/MDS, according to ECIL recommendations [29–31]. Additionally, modified transplant procedure for pretransplant IFD, such as no-TBI conditioning, RIC, or use of PB as a stem cell source, could have possibly decrease the rate of fungal reactivations [1, 32]. Proven IFD were more often diagnosed in children. This was due mainly because of diagnosis of candidemia, as children usually have permanent, while adults rather temporarily central venous catheters. Most of children were also receiving TPN, while it was rather infrequent practice in adults. Relatively high rate of candidemia among proven IFD in children probably contributed to lower IRM than in case of invasive aspergillosis, as reported recently [33].

The incidence of viral infections was higher in children than in adults. This observation can be explained by immature immune system in children, resulting in primary infection or higher rate of reactivation of latent viruses. CMV and EBV were two most often diagnosed viruses in children after allo-HCT. CMV exerts direct and indirect effects in tissues and often plays a role of driver of another infections, including IFD, thus contributing to an increased post-transplant risk of life-threatening complications. With respect to respiratory viral infections, there is no current strategy of routine monitoring of community-acquired respiratory viruses; thus, no firm conclusion can be drawn on this topic from our study.

IRM was higher in adults, what has been evidence-proved for the first time. Additionally, IRM was higher in Gram-negative infections and in patients with acute leukemia. The outcome of infections was better in children both after allo- and auto-HCT. In addition to well-defined factors for mortality (acute leukemia, MMUD, GVHD, CMV reactivation), duration of infection > 21 days was associated with an increased risk of death after infection.

The higher infection rate of MDI in children in comparison to adults can be explained by the following factors: (1) much higher rate of auto-HCT in adults resulting in overall lower incidence of infections in adults, especially seen in case of bacterial complications; (2) higher rate of patients with acute leukemia in pediatric cohort, with a well-known high incidence of infectious complications in acute leukemia [8, 34]; (3) much higher rate of diagnosis of possible IFD in children being the consequence of the positive results of imaging only; (4) higher incidence of viral infections in children, what can correspond to higher rate of primary infections; and finally, (5) real-life tendency of pediatricians to perform more detailed diagnostic procedures. Due to the same factors, the diagnosis of multiple myeloma was associated with a decreased risk for infection in multivariate analysis. On the other hand, the incidence of infectious complications in this group of patients was similar as presented in recent analyses [35–38].

The limitation of the study is its retrospective design; however, data were collected periodically. Also no routine screening was performed for viral infections except CMV and EBV. Thus, in most cases of viral infections, the diagnosis was bound to clinical symptoms.

In conclusion, the profile of infections and related deaths largely vary between children and adults. Our study proved age-dependent determinants of pediatric and adulthood profile of infectious complications after HCT: children have higher risk of all types of infections and a better outcome of bacterial infections, while in fungal and viral infections, the IRM was comparable between children and adults. Adult age, MMUD transplants, diagnosis of acute leukemia, chronic GVHD, CMV reactivation, and infection lasting > 21 days are relative risk factors for death from infection after HCT. The potential implication of this comprehensive analysis might be differential infection control and management strategies for children and adults.
